# Flexural Performance and Stress Calculation of External Prestressed Fiber-Reinforced Polymer-Bar-Strengthened One-Way Concrete Slabs

**DOI:** 10.3390/ma17051130

**Published:** 2024-02-29

**Authors:** Dong Fang, Danying Gao, Chong Ding, Zhiqiang Gu, Peibo You, Jiyu Tang

**Affiliations:** 1 School of Civil Engineering, Zhengzhou University, Zhengzhou 450001, China; fangdong8898@163.com (D.F.); gaodanyingzzu@126.com (D.G.); tjy74@zzu.edu.cn (J.T.); 2 School of Water Conservancy and Transportation, Zhengzhou University, Zhengzhou 450001, China; zqgzzu@163.com; 3 School of Civil and Transportation Engineering, Henan University of Urban Construction, Pingdingshan 467000, China; 30070513@huuc.edu.cn

**Keywords:** external prestressing, FRP bar, one-way slab, strengthened slab, flexural behavior

## Abstract

External prestressing is widely employed in structural strengthening engineering due to its numerous advantages. However, external prestressed steel bars are prone to corrosion when exposed to the service environment. This paper is dedicated to examining the use of fiber-reinforced polymer (FRP) bars as external prestressing materials to strengthen one-way concrete slabs. Five one-way concrete slabs were strengthened with externally prestressed FRP bars with different prestress levels and different amounts of FRP bars, while one non-strengthened slab was used for comparison. The effects of strengthening on the flexural behavior, specifically the cracking load, ultimate load, stiffness and failure mode, were analyzed systematically. Moreover, the ductility and cost–benefit optimizing properties of the reinforcing design were discussed. The results show that external prestressed FRP bars significantly improve the cracking load, ultimate load and stiffness of one-way concrete slabs. The absence of a bond between the concrete and FRP bars overcomes the brittleness of the FRP bars, while the strengthened slabs exhibit satisfactory ductility and a higher post-yield stiffness and bearing capacity. Additionally, the cost/benefit ratio is optimized by increasing the prestress level, while a higher number of prestressed FRP bars is beneficial to ductility. Finally, a method for calculating the stress in prestressed FRP bars at ultimate loads was proposed. Irrespective of the prestressing material, this method is applicable to both strengthened beams and one-way slabs.

## 1. Introduction

In recent years, many existing concrete structures throughout the world have deteriorated continuously to such a degree that strengthening is necessary to maintain a normal working service. External prestressing is an effective solution for improving the carrying capacity, reducing deformations and closing cracks [1,2]. Nevertheless, external prestressed steel bars are exposed to the service environment and thus suffer from corrosion. To overcome this shortcoming, fiber-reinforced polymer (FRP) bars, composed of carbon, basalt or glass fibers, have been developed and proffered as an alternative to conventional steel bars [3,4].

FRP bars have high tensile strength, lightweight and excellent corrosion resistance. However, FRP bars are characterized by significant brittleness, and their elastic modulus is lower than that of steel bars. These problems mean that concrete reinforcement with FRP bars tends to incur larger deflections, wider cracks and brittle failure under an external load [5]. Fortunately, using FRP bars in an external prestressing system can avoid the aforementioned deficiencies. Pre-tension stress in FRP bars confines cracks and deflection by offsetting a portion of the service load. Moreover, the absence of bond results in elongation. This is induced by bending deformations that are uniformly spread along the entire FRP bar, thereby decreasing the maximum strain. Therefore, the FRP bar remains unbroken even if the prestressed concrete has reached its limit [6]. In fact, the force transferred between the external prestressed bar and concrete depends on the anchors and deviators. As a result, a conventional cross-section analysis is no longer applicable. The strain of the external prestressed bar is determined by the relative displacement and the rotation of the anchors and deviators under an external load. In turn, the response of the strengthened member is affected by the stress in the external prestressed bar. Therefore, the flexural properties and analyses of strengthened members are complex [7]. Furthermore, the application of FRP bars as an alternative external prestressing material introduces more indefinite factors due to their special properties [8]. 

The calculation of stress in external prestressed bars plays an important role in flexural behavior predictions, in which a member analysis must be adopted. To simplify these calculations, Naaman [9] introduced a bond reduction coefficient to revise the unbonded effects during cross-section analyses, which is known as a pseudo-section analysis. On this basis, various flexural experiments and finite element simulations were conducted to investigate concrete beams prestressed with unbonded steel tendons [10,11,12,13]. Several equations concerning the bond reduction coefficient were presented. These equations consider the span/depth ratio (the ratio of span to prestressed tendon depth), deviator location, load distribution and the ratio of non-prestressing to prestressing reinforcement, each to varying degrees.

Tan [14] employed carbon FRP (CFRP) bars to strengthen a concrete T-beam with external prestressing. Beams that were externally prestressed using CFRP bars exhibited similar flexural properties to those prestressed with steel bars. Namaan [15] analyzed previous experiments on concrete beams externally prestressed with steel or FRP bars and presented a calculation method for stress increases in external prestressed bars. Moreover, a simplified equation, in which the elastic modulus of external prestressed bars was the only considered factor, was also proposed. Ghallab [16] conducted numerous flexural experiments on concrete beams externally prestressed with glass FRP (GFRP) parafil ropes, and a statistical analysis of previous studies was carried out to enhance the data coverage. The results demonstrated that the stress of the external prestressed bar is influenced by similar factors and follows similar laws, regardless of whether the material is steel or FRP. However, the effect was slightly weaker in the case of FRP due to its lower elastic modulus. Based on a deflection analysis, Ghallab [17] proposed a method to calculate the flexural capacity of strengthened continuous beams. Lou [18] presented a simplified equation to calculate the redistribution of strengthened continuous beams at the ultimate load. The research results also showed that the ductility of external prestressed concrete beams with GFRP bars was even better, even though their bearing capacity was lower than beams with CFRP or steel tendons [19]. Wang [20] discussed the effects of anchorages and the deviator bending angle on the prestress loss and strength reduction of basalt FRP (BFRP) bars. An optimized deviator design was applied to beams externally prestressed with BFRP bars, which exhibited satisfactory carrying capacity, crack patterns and ductility. Sun [21] applied external prestressing technology to alter a conventional FRP-bar-reinforced concrete beam, wherein certain commonly used FRP bars were pre-tensioned and utilized as external prestressed bars. As a result, the flexural carrying capacity, crack pattern and ductility improved significantly.

Previous studies have indicated that FRP bars are an attractive material for external prestressed systems. The flexural properties of concrete beams externally prestressed with FRP bars have been sufficiently investigated, and various calculation methods have been presented. However, one-way concrete slabs, as another common bending member, have scarcely been used as a strengthening member in previous studies. In practice, the characteristics of one-way slabs, such as the higher span/depth ratio, the lower reinforcement ratio and the unique distribution of prestressed bars, result in different flexural behavior compared with beams. In this study, a series of bending experiments on one-way concrete slabs strengthened with externally prestressed FRP bars were conducted. This study aims to explore how the number of prestressed FRP bars and the prestress level influence the flexural behavior of one-way slabs and to examine how their properties differ from beams. Moreover, the ductility of the strengthened slabs was discussed. Finally, a simplified calculation method for examining the stress in prestressed FRP bars at ultimate load was proposed based on plastic hinge deformation.

## 2. Experimental Program

**Figure 1:**
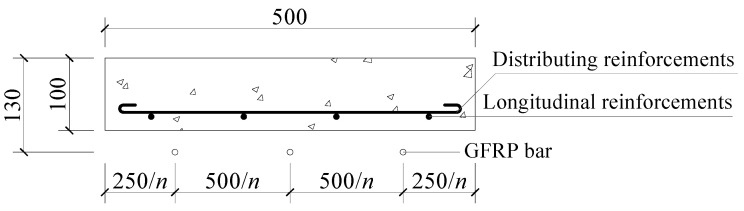
Dimensions and cross-section of a one-way slab (unit: mm). Note: *n* is the GFRP bar amount.

This paper focuses on the application of FRP bars as an external prestressing material in strengthened one-way concrete slabs. Six one-way concrete slabs with the same reinforcements were studied. Five slabs were strengthened with externally prestressed FRP bars with different prestress levels and different amounts of prestressed FRP bars, while the remaining slab was not strengthened and served as the “control specimen”. To simulate reinforced slabs, the axial compressive strength of concrete was chosen as 30 MPa, and the longitudinal reinforcements consisted of four D8 plain steel bars close to the minimum reinforcement ratio. In the vertical direction, D8 plain steel bars were used as distributing reinforcements. The steel bars’ yield strength and elastic modulus were measured as 319.3 MPa and 209.5 GPa, respectively. GFRP bars with a nominal diameter of 8 mm were selected as the external prestressed bars. The observed tensile strength and elastic modulus were 464.2 MPa and 43.2 GPa, respectively. A cross-section of the specimens is shown in Figure 1, along with the specific dimensions. 

**Figure 2:**
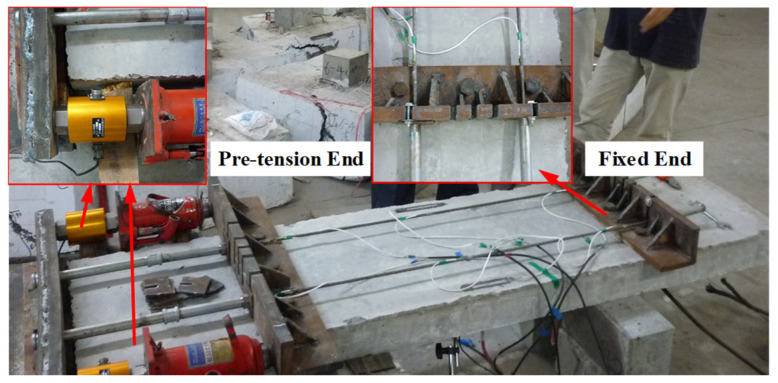
Anchoring and prestress tension of GFRP bars.

**Figure 3:**
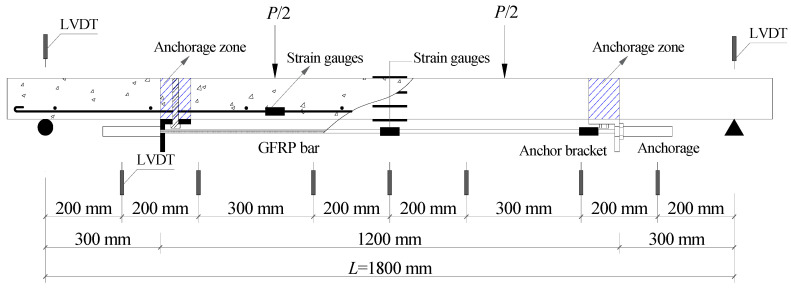
Specimen details and measurement point distribution. Note: *P* and *L* are the applied load and the net span, respectively.

The slabs were simply supported under their short sides. According to the size of the reaction frame, the length and net span of the slabs were 2000 mm and 1800 mm, respectively. The length/width ratio was greater than three, satisfying the conditions for one-way bending. At a length of 1200 mm, in mid-span, an area with larger bending moments, the slabs were strengthened with external prestressed GFRP bars, which were fixed onto the bottom of the slab using anchors and an L-shaped plate, as shown in Figure 2 and Figure 3. The interface of the L-shape plate and concrete is the anchorage zone to transfer prestress, and it had a length of 75 mm. Anchors were applied during the tensile tests of the GFRP bars, and all the GFRP bars were successfully broken due to tension.

**Figure 4:**
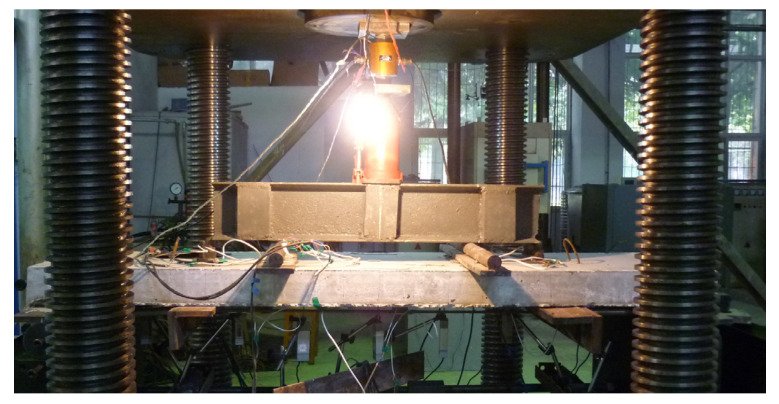
Specimen details and measurement point distribution.

Prestress was applied through a hydraulic jack, with the force being monitored and regulated by a load sensor, as in Figure 2. During pre-tensioning, the prestress was monitored and adjusted by using data from strain gauges. The prestress tension tests were divided into two steps. In the first step, the FRP bars were over-tensioned with stress values of up to 1.1 *f*_ed_ (*f*_ed_ is the default prestress level). After 24 h, the FRP bars were relaxed, and then a tension of 1.05 *f*_ed_ was applied again. Prior to loading the slab, the tensile stress in the FRP bars was adjusted to the designated prestress level. Throughout this process, strain values were continuously monitored and recorded every hour using strain gauges. The effective prestress in the FRP bars was calculated based on the elastic modulus and the average value of the measured strain. The details of the specimens, including the arrangement of strain gauges and linear variable differential transformers (LVDTs), are shown in Figure 3 and Table 1. The load was applied in steps of 1 kN via a hydraulic jack with a capacity of 50 kN, as shown in Figure 4. Each incremental load was sustained for at least five minutes to ensure complete crack and deformation development. Meanwhile, the strain in the concrete, steel and FRP bars was reset before loading and then measured by the data collector under external loads. All the slabs were loaded to failure via four-point bending. The flexural behavior and effects of external prestressed FRP bars were discussed and analyzed according to the experimental results. 

## 3. Results

**Figure 5:**
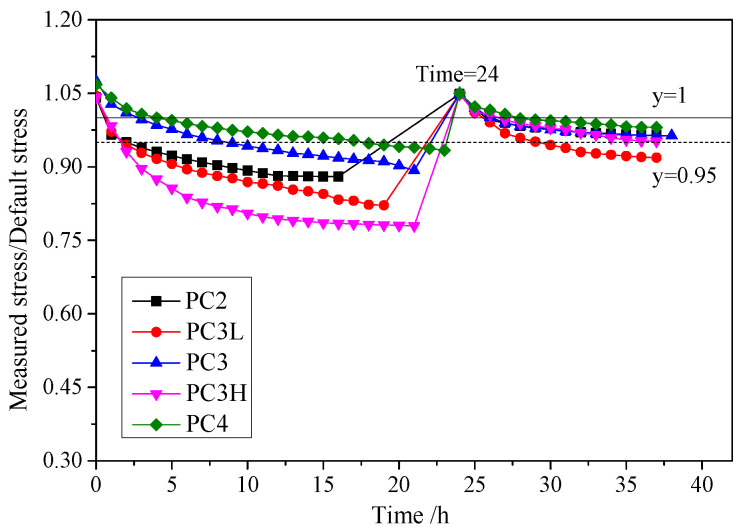
Relative effective prestress versus time.

The main experimental results are provided in Table 2, where *f*_pe_ and Δ*f*_p_ are effective prestress and stress increment in FRP bars at failure, respectively; *ε*_u_ is the compressive strain of concrete in the top fiber at failure; *P*_cr_, *P*_y_ and *P*_u_ are the loads at concrete cracking, reinforcement yielding and ultimate failure, respectively; Δ_y_ and Δ_u_ are the mid-span deflections corresponding to *P*_y_ and *P*_u_, respectively; and *P_L_*_/300_ denotes the load of the mid-span deflection equal to *L*/300. The variation in stress in the FRP bar was recorded after prestressing. The ratio of measured stress to the default prestress level with time is shown in Figure 5. At the moment of release, each specimen experienced a prestress loss of approximately 5%, with the majority of the loss occurring within the first 12 h after prestressing. Specimen PC3H exhibited a greater loss in prestress compared with the other specimens due to its higher initial prestress level. However, the prestress loss of specimen PC3L was only lower than that of PC3H. The most likely reason for this is that the anchor system contained defects caused during production or installation. Anchorage failure led to the strengthening function being lost in the external FRP bars in specimen PC3L. During the first 24 h, some of the slippage between the GFRP bars, anchorages and concrete was eliminated, and some small gaps were closed. As a result, the prestress loss significantly decreased after tensioning again. The effective prestress remained higher than 95% in all specimens except for PC3L. 

**Figure 6:**
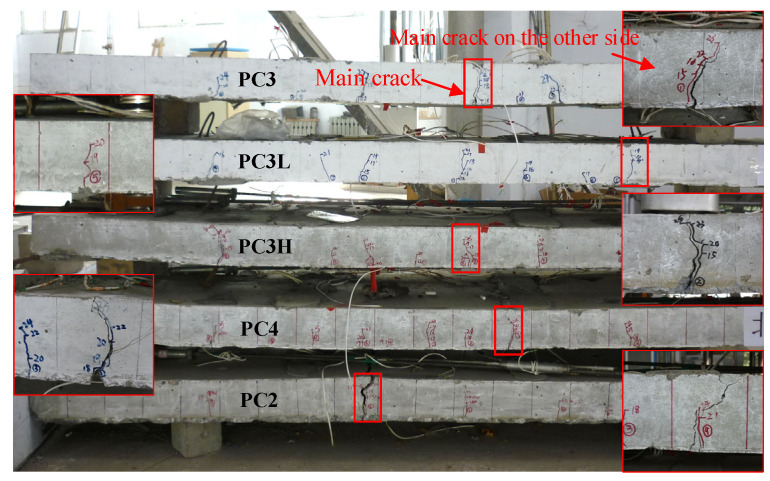
Crack and flexural failure pattern.

The failure forms and crack patterns of the strengthened slabs are shown in Figure 6, in which major cracks and the corresponding cracks on the other side are framed in red. Due to the insufficient bearing capacity, the control slab experienced rapid and severe damage after the reinforcements yielded. As a result, the entire specimen was not retained after failure. Specimen PC2 exhibited more severe damage due to its lower FRP bar content. Specifically, the anchorage zone of slab PC3L cracked prematurely, and this crack led to anchorage failure. Therefore, PC3L was unable to be used to analyze the bending behavior of the strengthened one-way slab. In contrast, the cracks in specimen PC4 were narrower but more numerous. The remaining specimens had similar crack patterns. During the experimental process, only one or two primary cracks gradually extended, nearly spanning the thickness of the slab, with a minimal amount of concrete being crushed. The final failure mode was similar to that of rare-reinforced beams in appearance. However, a much higher load-carrying capacity still remained after the steel bars yielded, in stark contrast to rare-reinforced beams. The effects of the improvements to these specimens were various due to the different amounts of prestressed FRP bars and the different prestress levels and are discussed in the following.

## 4. Discussion

### 4.1. Effect of Prestressed FRP Bar Amount

The cracking loads of specimens PC2, PC3 and PC4 were improved by 24%, 81.9% and 121%, respectively, with respect to RC. For the yielding loads, corresponding increases of 8.8%, 74.0% and 67.9% were observed, and for the ultimate load, these increases were 38.6%, 79.9% and 101.5%. In general, greater strengthening effects were achieved by incorporating prestressed FRP bars, with these bars assuming distinct roles from the initial loading stage to the ultimate capacity state. Owing to the absence of bonds, the FRP bars effectively constrained concrete cracking through the exertion of reverse bending moments, thereby mitigating tensile stress in the concrete zone. Before cracking, the applied load and corresponding deformations were small, and the prestressing force offset most of the applied load. Under the ultimate load, the tension zone of the slab had no carrying capacity, and almost all the incremental load was borne by the external prestressed FRP bars. Therefore, the external prestressed FRP bars performed an important role in these two stages. The improvements in the cracking load and ultimate load were almost linearly related to the amount of prestressed FRP bars. 

**Figure 7:**
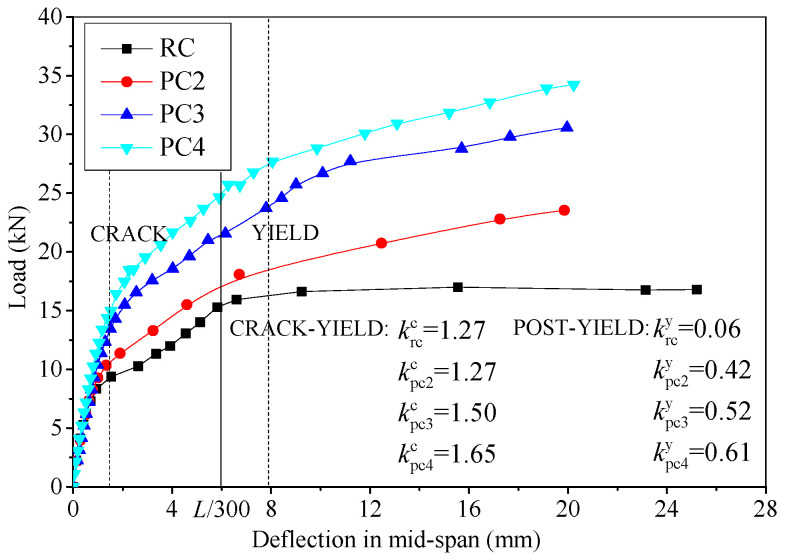
Load–deflection curves for specimens with different amounts of FRP bars.

As mentioned above, the mechanical mechanisms of the two critical events, concrete cracking and steel bar yielding, changed, while the responses of the specimens under an applied load can be divided into three stages. The load–deflection curves for slabs with different amounts of FRP bars are shown in Figure 7. Before concrete cracking, the slabs demonstrated comparable stiffnesses due to the relatively limited quantity and elastic modulus of the FRP bars. The enhancement in stiffness due to the FRP bars became more apparent after concrete cracking. During the crack-yield and post-yield stages, the average slopes of the curves were calculated to determine the stiffness and carrying capacity of load increment, as shown in Figure 7 (*k*^c^ and *k*^y^ are the average slopes of crack-yield and post-yield, respectively). In the crack-yield stage, the FRP bars assisted the steel bars to bear the external load, which improved the stiffness of the strengthened slabs. However, the *k*^c^ values of PC2 and RC were equal because the amount of external prestressed FRP in PC2 is lower. The *k*^c^ values of PC3 and PC4 were 18% and 28% higher than those of PC2 due to the increase in the amount of FRP bars. When the mid-span deflection reached the serviceability limit, *L*/300, the corresponding loads of the strengthened slabs PC2, PC3 and PC4 improved by 14.1%, 41.4% and 61.9%, respectively. After the steel bar yielding, the *k*^y^ of RC was almost zero, while the *k*^y^ values of PC2, PC3 and PC4 increased from 0.42 to 0.61. This is because the FRP bars in strengthened slabs resisted higher loads and contributed to the post-yield stiffness. Thus, employing more prestressed FRP bars resulted in an increased stiffness, an improved serviceability limit capacity and enhanced safety reserves for the strengthened slabs. 

**Figure 8:**
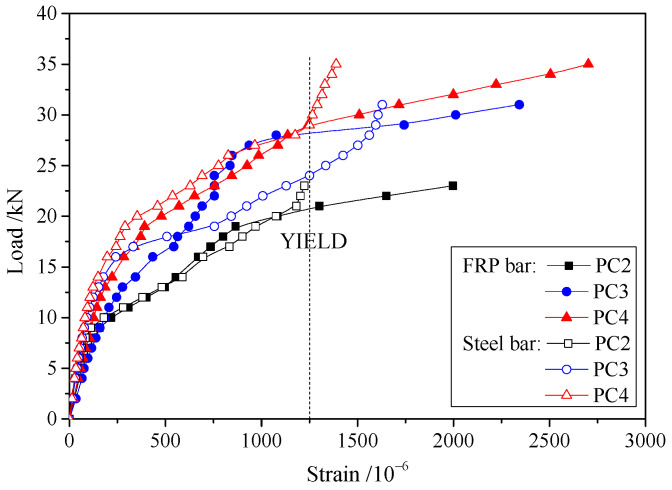
Load–strain curves for specimens with different amounts of FRP bars.

The compressive strains of concrete at failure in the top fibers are shown in Table 2, while the average values of the measured strain in FRP and steel bars are shown in Figure 8. With fewer prestressed FRP bars, each steel and FRP bar is required to bear a greater amount of stress in the strengthened slab. Therefore, as the quantity of prestressed FRP bars decreases, the strains in the steel and FRP bars of the strengthened slab increase. For prestressed FRP bars, the general trend in the load–strain curve is similar to that in the load–deflection curve. This highlights the close correlation between the strengthening effects of FRP bars and slab deformation. The load–strain curves of steel bars exhibited a rapidly ascending stage after yielding. The reason for this is that steel bar yielding only occurred in the cracks across segments, while the plastic hinge deformed only around the main cracks. As a result, the strain of the steel bar barely increased in other segments, and the additional load was borne by the FRP bars. This confirms that the strengthening effects of the FRP bar are pronounced from an alternate perspective. Furthermore, the FRP bar prevents the strengthened slab from collapsing entirely.

### 4.2. Effect of Prestress Level

The strengthened slabs PC3L, PC3 and PC3H, containing the same FRP bar amount but with different prestress levels, reached 42.1%, 81.7% and 51.4% higher cracking loads, respectively, than RC because the tensile stress of concrete in the tension zone was counteracted by the reverse bending from the external prestressed FRP bars. In general, the anti-cracking ability improved significantly, especially in slabs with higher prestress levels. Nevertheless, for specimen PC3L, the flexural cracks generated under the load point were followed by cracking near the anchorage. Furthermore, the cracks near the anchorage widened more severely with an increase in the applied load. As a result, the interaction of the FRP bars and concrete deteriorated significantly, and the stress increment in the FRP bar at failure was just 27 MPa. This could potentially be attributed to the lack of precision in anchor installation or the inadequate compactness of the concrete in the anchorage zone. In the phase of prestressing application, substantial prestress loss may also indicate underlying problems with the anchors. The specimen PC3H, with a higher prestress level, exhibited a 69.5% improvement in the ultimate bearing capacity over RC. However, this capacity was still slightly lower than that of PC3 due to the lower stress in the FRP bar at the ultimate load.

**Figure 9:**
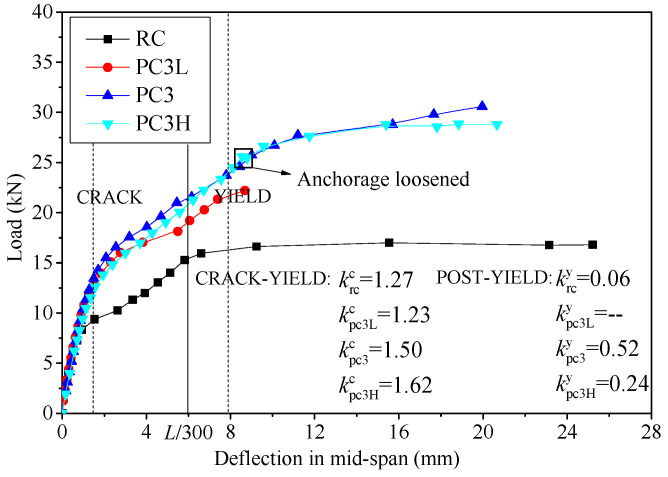
Load–deflection curves for specimens with different prestress levels.

The load–deflection curves of specimens with different prestress levels are shown in Figure 9, along with their average slopes at different stages. Generally, the stiffness of these slabs is expected to be similar due to the identical amounts of FRP bars used. Nevertheless, the *k*^c^ of PC3H is much greater than that of other specimens. One potential explanation for this is that the elevated prestress level minimized the damage to the concrete in the tension zone, while the uncracked concrete exhibited a more pronounced hardening effect. After yielding, the anchorage failed quickly for slab PC3L, which resulted in the absence of a post-yield stage. In addition, slight loosening occurred in the anchorage of PC3H, and a small fluctuation in the load–deflection curve was observed. As a result, the *k*^y^ was only half of that of slab PC3. This also provides an explanation for why the stress increment in the FRP bar was lower than that of PC3 at the ultimate load.

**Figure 10:**
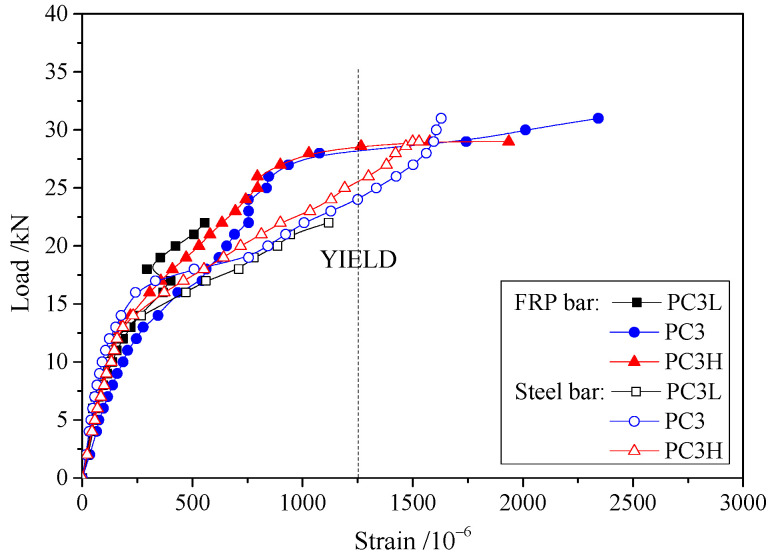
Load–strain curves for specimens with different prestress levels.

The load–strain curves for FRP and steel bars with different prestress levels are shown in Figure 10. The curves exhibit similar trends to those in Figure 8. However, the strain in the FRP and steel bars has no obvious variation with different prestress levels. Hence, the stress in external prestressed FRP bars is not influenced by the prestress level, and it was not taken into account during Δ*f*_p_ calculations.

In fact, the prestress in the FRP bars partially offsets the external load and reduces the deflection by generating a negative moment. Furthermore, the external prestressed FRP bars bear the external load in combination with the slab, resulting in an enhanced carrying capacity and stiffness. Therefore, high prestress levels will improve the cost/benefit ratio in anti-crack designs. However, further research is warranted to investigate the long-term prestress loss and the reliability of the anchorage system in external prestressed FRP bars under high prestress levels.

### 4.3. Ductility

The energy ductility factor serves as a comprehensive measure of both the load-carrying capacity reserve and the energy dissipation capability and is used to quantitatively represent the ductility of each specimen. The energy ductility factor equation is expressed as follows:

*D*_c_ = *A*_su_/*F*_s_Δ_s_, (1)

where *D*_c_ is the energy ductility factor; *F*_s_ is the applied load in the service state; Δ_s_ is the deflection corresponding to *F*_s_; and *A*_su_ is the area under the load–deflection curve from the service state to the ultimate limit state.

*P_L_*_/300_ was chosen as the service load, while the yielding load was less than *P_L_*_/300_ and was chosen as a service load for specimens PC2 and PC3L. In this case, energy ductility factors were calculated and are shown in Table 2. In particular, the failure of specimen PC3L was caused by anchorage invalidation, which resulted in a small post-yield stage and much lower ductility. Compared with the control specimen, RC, the strengthened slabs exhibited ductile failure on the whole, and their energy ductility factors were reduced by no more than 15%, despite the fact that FRP bars are often considered brittle materials. The reasons for this are that the prestressed FRP bars were only connected to the strengthened slab via each end of the anchorage, and the deformation was evenly distributed throughout the FRP bar. In other words, the strain of the FRP bar is affected by the deformation state of the entire member. The external prestressed FRP bars do not exhibit tension failure at the ultimate limit state. Therefore, the absence of a bond between the concrete and the FRP bar overcomes the brittleness of the FRP bar, although the bearing capacity and stiffness are reduced. 

Overall, the energy ductility factor is negatively correlated with the stress in the FRP bar at ultimate loads. For slabs PC2, PC3 and PC4, the energy ductility factor was reduced by 14% when the FRP bars increased from two to four. One reason for this is that the *P*_u_-to-*P*_y_ ratio of PC2 is close to 1.4. This indicates that PC2 has a higher bearing capacity reserve. Another possible cause is that the higher amount of FRP bars improved the load in the service state, while the ultimate deflections were similar. Analogously, a higher prestress level reduces deformability and ductility. However, the slab PC3H had a higher energy ductility factor than PC3 due to the lower stress in the FRP bar at ultimate loads. This also indirectly confirms that the loss in ductility is unavoidable when enhancing the bearing capacity in an externally prestressed strengthening system. The application of FRP bars in this system preserves an acceptable ductility for the strengthened members, while the strength and durability of FRP bars are exploited.

External prestressed FRP bars provide an adequate post-yielding carrying capacity and deflection for the strengthened slab, though their energy ductility factor is lower than non-strengthened slabs. Moreover, the integrity and safety of the reinforced slab are significantly enhanced, thereby preventing the total collapse of the slab. In addition, FRP bars, as a linear elastic material, effectively enhance resilience. It is worth noting that the ultimate carrying capacity of PC4 is 12% higher than PC3, though their energy ductility factors are similar. Therefore, increasing the amount of FRP bars is a crucial method for improving bearing capacity, especially when the energy ductility factor is constrained by design specifications. However, the cost/benefit ratio is optimized by increasing the prestress level and reducing the FRP bar amount on the condition that the requirements for ductility and FRP bar strength are satisfied.

## 5. Calculation of Stress in Prestressed FRP Bars

### 5.1. Previous Calculation Method

The principal problem in bearing capacity predictions is the calculation of the stress in the external prestressed FRP bars. By an analysis of the test results, it was shown that the stress increment in FRP bars depends on the deformation state of the whole member. The traditional calculation method, based on the strain compatibility in the cross-section, is no longer applicable. To solve this problem, Naaman [15] developed a “pseudo-section analysis” method, in which the effects of the deformation of the entire member were summarized in a bond reduction coefficient. The stress in external prestressed bars can be calculated as follows:

(2) $$f_{p}=f_{\mathrm{pe}}+\Omega E_{p}\varepsilon_{c}(\frac{d_{p}}{c}-1),$$

where *f*_p_ and *E*_p_ are the stress and elastic modulus of the external prestressed bar; *ε*_u_ is the compressive strain of concrete in the top fiber; *d*_p_ is the effective depth of the external prestressed bar; *c* is the depth of the neutral axis; and Ω is the bond reduction coefficient.

The stiffness and material properties of concrete members continuously change under various loads because of concrete cracking or steel bar yielding. For this reason, Naaman [15] proposed the bond reduction coefficient calculation method for use under various loads and material states. However, this method is very complex and difficult to apply. Indeed, the calculation requires knowledge of the ultimate state of the member in most cases. According to previous research, the stress increment in external prestressed bars is influenced by several factors, such as anchorage location, span/depth ratio, deviator location and load distribution. Some methods for calculating the bond reduction coefficient at ultimate loads, in which several of the above factors were taken into account, have been proposed, as shown in Table 3. 

### 5.2. Simplified Calculation Equation

**Figure 11:**
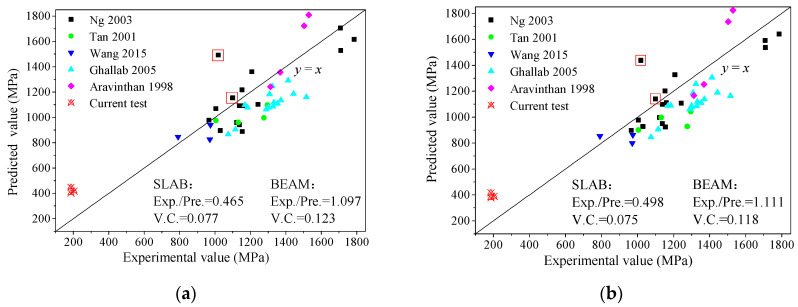
Evaluation of previous prediction methods: (**a**) Naaman; (**b**) Aravinthan [13,14,16,20,22].

A set of 43 specimens from previous research was collected, and some of their parameter ranges are shown in Table 4. The stress in external prestressed bars at failure was calculated by the equations in Table 3 and compared with the experimental values. The values calculated via Naaman’s [15] and Aravinthan’s [22] methods are close to the experimental values, with a slight tendency towards being conservative, as shown in Figure 11. On average, the ratios between the experimental and predicted values (Exp./Pre.) are equal to 1.097 and 1.111, respectively. In addition, their variable coefficients (V.C.s) are equal to 0.123 and 0.118, respectively, which suggests that these equations are quite robust. For concrete beams externally prestressed with BFRP or GFRP bars, the majority of calculated values (using Wang’s [20] and Ghallab’s [16] methods) exhibit a slight discrepancy from the corresponding experimental values, with the former being marginally smaller. Therefore, these two calculation methods are suitable for concrete beams externally prestressed with FRP bars. However, the results showed that the predicted values are not in agreement with the experimental values determined in this work. This indicates that the characteristics of the slabs, such as the higher span/depth ratio, lower reinforcement ratio and unique failure form, must be taken into account. In addition, these equations are tedious and must be solved by simultaneous equations with section force balance. 

However, for example, the FRP bars in specimen PC3 provided about 40% of the bearing capacity at the ultimate load, while 60% of the stress in the external prestressed FRP bar originated from the elongation of the FRP bar after loading. Consequently, it was determined that the deviation in the computed incremental stress in FRP bars would not notably impact the bearing capacity calculation accuracy. Therefore, it is not cost-effective to spend a lot of computing resources [15]. Using another method based on the plastic hinge length can simplify the calculation process, though the calculation is not very accurate. In this method, the elastic deformation of the member is neglected, and only the elongation of the external prestressed bars, derived from the plastic hinge deformation, is considered. The stress in the external prestressed bar can be calculated as follows:

(3) $$f_{\mathrm{pu}}=f_{\mathrm{pe}}+\frac{E_{p}}{L_{s}}\varepsilon_{\mathrm{cu}}\frac{d_{p}-c}{c}L_{p},$$

where *L*_s_ and *L*_p_ are the external prestressed bar length and the plastic hinge length, respectively.

Lee [24] proposed that the plastic hinge length of the concrete beam can be taken as the constant moment segment, with the addition of the effective section depth, as follows:

(4) $$L_{p}=L_{0}+2\times 0.5h_{0}$$

**Figure 12:**
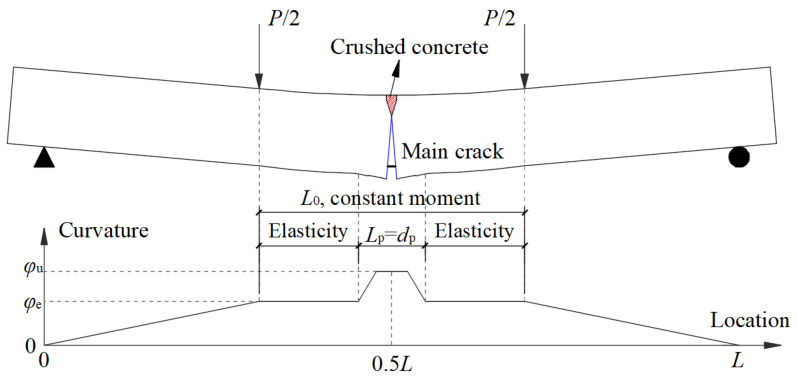
Elastic and plastic deformation of a concrete one-way slab.

However, the concrete was crushed only above one or two cracks at failure in the tested one-way slabs, while the residual concrete in the compressive zone did not fail according to the experimental results (as shown in Table 2). For this reason, the calculated results from the equations in Table 3 were much larger than the experimental values of *f*_p_ for the slabs. In this case, plastic deformation occurs primarily around the main cracks, as shown in Figure 12. The majority of the constant moment zone remains in an elastic phase, and the elongation of the external prestressed bars in this segment should not be neglected. The neutral axis in the elastic segment is about half of the slab thickness. The stress in the external prestressed bar can be calculated as follows:

(5) $$f_{p}=f_{\mathrm{pe}}+E_{p}(\varepsilon_{\mathrm{cu}}\frac{d_{p}-c}{c}\frac{L_{p}}{L_{s}}+\varepsilon_{u}\frac{2d_{p}-h}{h}\frac{L_{0}}{L_{s}})$$

To simplify the calculation, the plastic hinge length and compressive strain of concrete in the top fiber at failure were estimated as *h*_0_ and 0.5*ε*_cu_, respectively. Herein, Equation (5) was further simplified, and a simple equation for the evaluation of stress in the FRP bar was deduced as follows:

(6) $$f_{\mathrm{pu}}=f_{\mathrm{pe}}+E_{p}\varepsilon_{\mathrm{cu}}(\frac{d_{p}}{25\rho_{p,s}}+\frac{L_{0}}{2})/L$$

where *ρ*_p,s_ is the total ratio of longitudinal reinforcements to prestressed bars.

**Figure 13:**
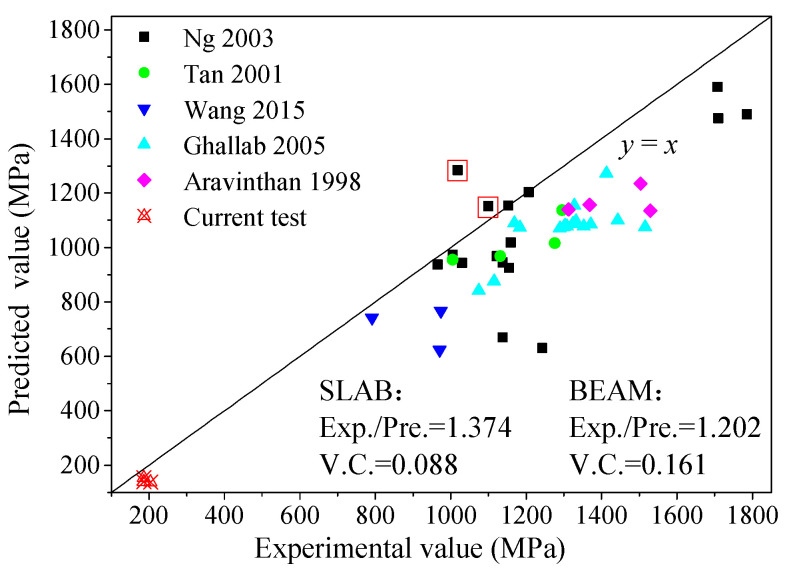
Comparison of experimental and predicted values [13,14,16,20,22].

It is worth noting that three crucial factors, including the elastic modulus of the prestressed bar, the ratio of longitudinal reinforcements and the elastic deformation in the constant moment zone, were taken into account in addition to the plastic hinge deformation. Therefore, irrespective of the prestressing material, this equation is applicable to both strengthened beams and one-way slabs. The *f*_pu_ values of the specimens in Table 4 and those determined in experiments in this paper were predicted. A comparison of the experimental and calculated values is shown in Figure 13. The results show that the stress values from Equation (6) are conservative and stable. Values that were higher than the experimental values were predicted only for 2 of the 47 specimens. In fact, higher predicted values were also produced by the equations in Table 3 for these two specimens (shown in Figure 11 and Figure 13). This may be attributed to experimental deviations. Therefore, it is feasible to evaluate the stress at failure in external prestressed bars using Equation (6) for concrete beams or one-way slabs externally prestressed with steel or FRP bars.

## 6. Conclusions

In this study, one-way concrete slabs were strengthened using external prestressed FRP bars to examine the feasibility of using them as external prestressing materials. The effects of the number of prestressed FRP bars and the prestress level on the flexural behavior, including bearing capacity, stiffness, ductility and failure mode, were discussed. In addition, a simplified calculation method for the stress in prestressed FRP bars at ultimate loads was proposed. The results highlight that this is a highly durable and efficient strengthening technique for one-way concrete slabs. The major conclusions are as follows:

(1) The strengthened slabs exhibited an improvement of over 100% in terms of the cracking resistance and ultimate load despite the low ratio of external prestressed FRP bars at only 0.4%. The experiments substantiated the efficacy, reliability and cost-effectiveness of this strengthening technique. Further investigations are required to examine the long-term prestressing loss and enhance the reliability of the anchorage system, particularly under high prestress level conditions.
(2) The strengthening effects of external prestressed FRP bars are attributable to two factors: the prestress in the FRP bars offsetting a portion of the applied load and the FRP bars sharing the external load with the steel reinforcements. The cracking load, ultimate load and stiffness of the strengthened slabs were improved significantly with increases in the number of prestressed FRP bars and the prestress level. In practice, the cost/benefit ratio can be optimized by increasing the prestress level and reducing the amount of FRP bars under the condition that the requirements for ductility and strength are satisfied.
(3) The absence of a bond between the concrete and the FRP bar overcomes the brittleness of the FRP bar. The strengthened slabs have satisfactory ductility and higher post-yield stiffness and bearing capacity, and their integrality, safety and resilience are significantly improved. Increasing the number of FRP bars is a crucial method for enhancing the bearing capacity, especially when the energy ductility factor is constrained by the design specifications.
(4) A method for calculating the stress in prestressed FRP bars at the ultimate load was proposed based on the plastic hinge deformation. Irrespective of the prestressing material, this method is applicable to both strengthened beams and one-way slabs. However, further investigations are required in the future to explore the effects of certain factors, including the deviator location and span/depth ratio, as the number of specimens in this research was limited.

## Figures and Tables

**Table 1:** Details of specimens under investigation.

Specimen ^1^	Reinforcement		*h*_0_ ^2^ (mm)	FRP Bar			Prestress Level, *f*_ed_ (MPa)	Concrete Property ^3^		
	Area (mm^2^)	Spacing (mm)		Diameter (mm)	Amount	Area (mm^2^)		*f*_c_’ (MPa)	*f*_t_ (MPa)	*E*_c_ (GPa)
RC	201	125	81	--	0	0	——	32.8	2.47	34.56
PC2	201	125	81	8	2	100	80	32.4	2.91	37.32
PC3L	201	125	81	8	3	151	60	34.8	2.98	36.58
PC3	201	125	81	8	3	151	80	34.8	2.99	33.58
PC3H	201	125	81	8	3	151	100	31.0	2.99	35.02
PC4	201	125	81	8	4	201	80	37.4	3.64	33.36

^1^ The number represents the FRP bar amount; “L” and “H” represent prestress levels of 60 MPa and 100 MPa, respectively. ^2^ *h*_0_ is the effective height of the cross-section. ^3^ *f*_c_’, *f*_t_ and *E*_c_ are the axial compressive strength, tensile strength and elasticity modulus, respectively.

**Table 2:** Experimental results for one-way slabs.

Specimen	*f*_pe_ (MPa)	Δ*f*_p_ (MPa)	*ε*_u_ (10^−3^)	*P*_cr_ (kN)	*P*_y_ (kN)	Δ_y_ (mm)	*P_L_*_/300_ (kN)	*P*_u_ (kN)	Δ_u_ (mm)	*D*_c_
RC	--	--	1.10	8.35	15.94	6.61	15.30	16.99	25.21	3.50
PC2	77.6	108.3	1.28	10.35	17.35	5.12	17.45	23.55	19.88	3.43
PC3L	55.2	27.0	0.95	11.87	17.04	3.84	19.20	22.20	8.68	1.45
PC3	77.2	126.2	1.40	15.21	27.73	9.01	21.38	30.58	19.95	2.98
PC3H	95.3	90.9	1.38	12.64	22.24	8.81	20.87	28.79	20.66	3.18
PC4	78.6	118.7	1.43	18.45	26.77	8.05	24.77	34.23	20.25	2.94

**Table 3:** Calculation methods of the bond reduction coefficient at ultimate loads.

Method Source	Considered Factors	$\mathrm{Equations}\;\mathrm{of}\;\Omega_{u}$ $\;\mathrm{for}\;f_{\mathrm{pu}}=f_{\mathrm{pe}}+\Omega_{u}E_{p}\varepsilon_{\mathrm{cu}}(\frac{d_{p}}{c}-1)$
Ng [13]	■ Deviator location ■ Load distribution	$\Omega_{u}=\frac{d_{p}}{h}(0.895-1.364\frac{a}{L})-K$. For $S_{d}/d_{p}\leq 15$,$K=0.0096S_{d}/d_{p}$; for $S_{d}/d_{p}>15$, K=0.144.
Naaman [15]	■ Span/depth ratio ■ Load distribution	For uniform or third-point loading, $\Omega_{u}=3/(L/d_{p})$. For one-point mid-span loading, $\Omega_{u}=1.5/(L/d_{p})$.
Aravinthan [22]	■ Prestressed bar amount ■ Span/depth ratio ■ Deviator location ■ Load distribution	For single-point loading, $\Omega_{u}=\frac{0.21}{L/d_{p}}+0.04\frac{A_{p,\mathrm{int}}}{A_{p,\mathrm{tot}}}+0.04$. For loading at the third points, $\Omega_{u}=\frac{2.31}{L/d_{p}}+0.21\frac{A_{p,\mathrm{int}}}{A_{p,\mathrm{tot}}}+0.06$.
Mutsuyoshi [23]	■ Span/depth ratio ■ Deviator location ■ Load distribution	$\Omega_{u}=\frac{1.47+10.3(L_{0}/L)}{L/d_{p}}-0.29\frac{L_{0}}{L}\frac{S_{d}}{L}$

Here, *f*_pu_ is the stress in prestressed bars at the ultimate load; Ω_u_ is the bond reduction coefficient at the ultimate load; *ε*_cu_ is the ultimate compressive strain of concrete in the top fibers; *a* is the distance from the support to the loading point; *S*_d_ is the distance between deviators; *L* is the effective span; *L*_0_ is the distance between symmetrically applied concentrated loads; and *A*_p,int_ and *A*_p,tot_ are the internal prestressed bar area and the total prestressed bar area, respectively.

**Table 4:** Parameter ranges of collected specimens.

Source	No. of Specimens	No. of Deviators	Section Form	Material of External Bar	*A*_p_ ^1^ (mm^2^)	*A*_s_ ^1^ (mm^2^)	*f*_c_*’* ^2^ (MPa)	*L* (mm)	*L/d*_p_ ^3^	*L*_0_*/L* ^3^
Ng [13]	16	0; 1; 2; 3	T-beam	steel	265	402	25~36	1500~6000	7.5~30	0.33
Tan [14]	3	0; 1	T-beam	steel	201	402	28	4500	22.5	0.33
	1	0	T-beam	CFRP	152	982	38	4500	22.5	0.33
Wang [20]	3	0; 2	T-beam	BFRP	226	760	32.4	5700	12.6; 15.2	0.33
Ghallab [16]	16	2	I-beam	GFRP	61	101	35~63	1800~3600	12.7~25.3	0.33
Aravinthan [22]	4	2	T-beam	steel	139~277	236	35	5200	13.9~20.8	0.33

^1^ *A*_p_ and *A*_s_ are the areas of external prestressed bars and internal non-prestressed steel bars, respectively. ^2^ *f*_c_’ is the axial compressive strength. ^3^ *d*_p_ is the effective depth of the external prestressed bar, and *L* and *L*_0_ are the effective span and distance between symmetrically applied concentrated loads, respectively.

## Data Availability

Data are contained within the article.
